# P21 activated kinase 2 promotes pancreatic cancer growth and metastasis

**DOI:** 10.3892/ol.2020.11950

**Published:** 2020-08-05

**Authors:** Guo-Wang Yao, Jing-Rui Bai, Da-Peng Zhang

Oncol Lett 17: 3709-3718, 2019; DOI: 10.3892/ol.2019.10040

Subsequently to the publication of this article, the authors have realized that the incorrect immunohistochemical images were selected for Fig. 5C, which was intended to show a comparison of the protein expression levels of P21-activated kinase 2 (PAK2) comparing between control mice and those transfected with PAK2 shRNA-transfected tumors.

The corrected version of Fig. 5, including the correct immunohistochemical data for Fig. 5C, is shown opposite. Note that the alterations made to this Figure do not affect the results or the conclusions reported in this paper, and all the authors agree to this correction. The authors thank the Editor for presenting them with the opportunity to publish this Corrigendum, and apologize to the Editor and to the readership of the Journal for any inconvenience caused.

## Figures and Tables

**Figure 5.**
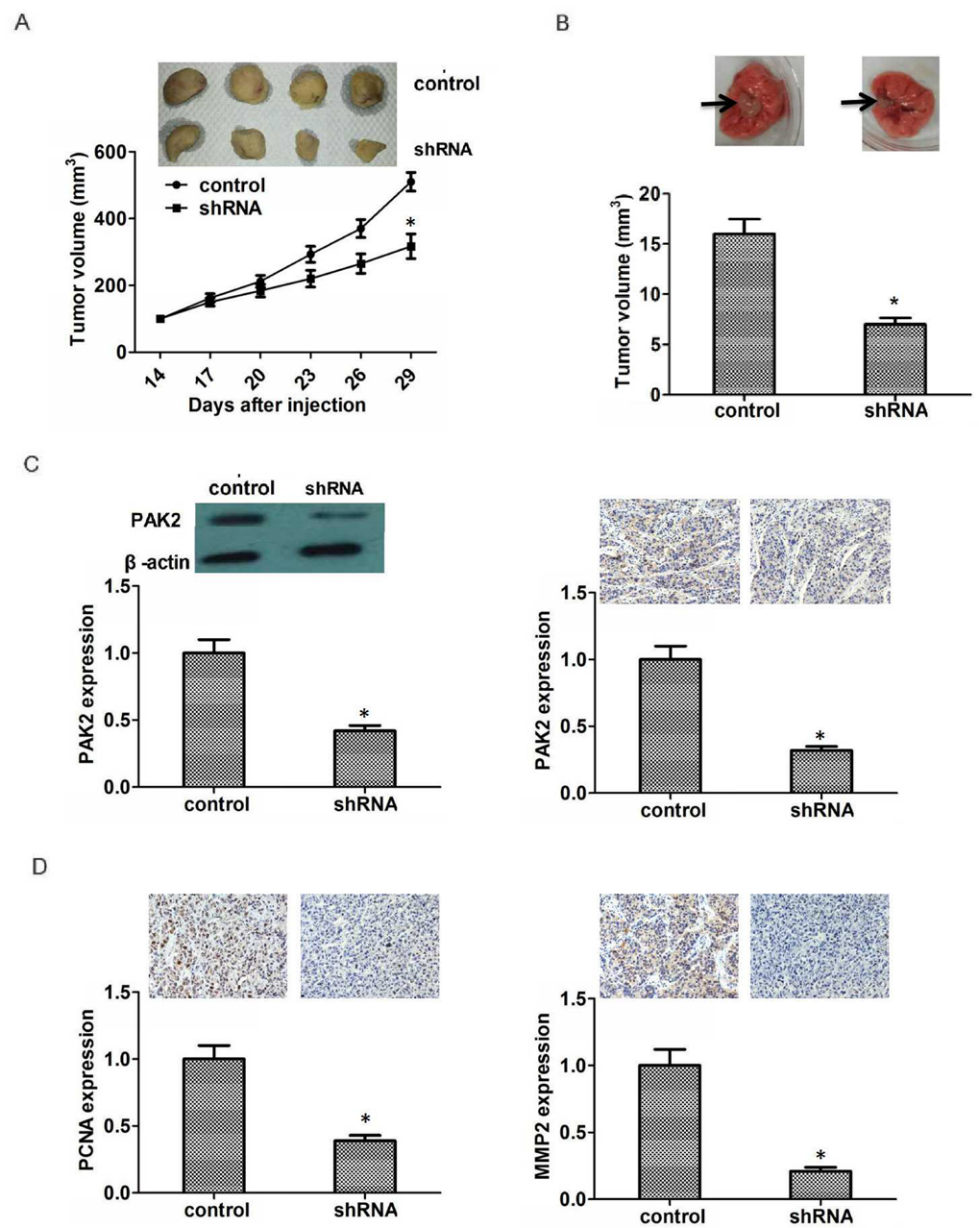
PAK2 promotes pancreatic cancer growth in mice. (A) PAK2-shRNA- and scramble shRNA-transfected PANC1 cells were injected subcutaneously into athymic nude mice and tumor volumes were measured every 3 days. Images were captured on the last day. (B) Lung metastatic tumors were harvested after 4 weeks of feeding. Tumors in the PAK2-shRNA group were smaller compared with the control group. (C) Protein expression level analysis of PAK2 in the two groups using western blotting and immunohistochemistry. (D) Protein expression level analysis of PCNA and MMP2 for the two groups using immunohistochemistry. Magnification, ×200. Data are reported as the means ± standard deviation (n=3). Arrows show lung tumor. *P<0.05, compared with the control. MMP2, matrix metallopeptidase 2; PAK2, P21 activated kinase 2; PCNA, proliferating cell nuclear antigen; shPAK2, short hairpin RNA specific to PAK2; shRNA, short hairpin RNA.

